# CAR-NK cell therapy for hematological malignancies: recent updates from ASH 2022

**DOI:** 10.1186/s13045-023-01435-3

**Published:** 2023-04-07

**Authors:** Ruihao Huang, Qin Wen, Xi Zhang

**Affiliations:** 1 Medical Center of Hematology, Xinqiao Hospital. State Key Laboratory of Trauma, Burns and Combined Injury, Army Medical University, Chongqing, China; 2 Jinfeng Laboratory, Chongqing, China

**Keywords:** CAR NK cell therapy, AML, MM, mAb

## Abstract

Chimeric antigen receptor (CAR)-NK cell therapy has the advantages of a low incidence of side effects and a low cost. However, the clinical outcomes are not satisfactory due to limited antitumor effects and a limited proliferative capacity. Recently, progress in CAR-NK cell therapy has been made in NK cell engineering, target design and combination with other agents to treat relapsed or refractory hematological malignancies, especially acute myeloid leukemia and multiple myeloma. This correspondence summarizes the preclinical and clinical updates for universal CAR-NK cell therapy reported at the ASH 2022 annual meeting.

To the editor:

Chimeric antigen receptor (CAR)-T cell therapy has substantially improved the outcomes of patients with hematological malignancies. However, insufficient autologous T cells, an extensive manufacturing time, severe side effects and a high price have restricted the clinical use of CAR-T cells [1]. CAR-NK cell therapy could be a universal, well tolerated and affordable treatment [2]. This report summarized the latest updates at the ASH 2022 annual meeting on the methods to improve the efficacy of CAR-NK cells.

## Preclinical studies

A new CAR screening platform was established to select an appropriate CAR transmembrane domain and endo-domain from 44 CAR constructs containing NK cell activating receptors, cytokine receptors and integrins to match NK cells through coculture target cell killing assays. The selected structure was tested in several cell lines, including SUP-B15, MOLT-4, and Raji, with different binding antigens and showed improved (more than 20%) antitumor efficacy in all killing assays compared with the reported NKG2D-2B4-CD3ζ CAR structure (Abstract 1983) [3]. FT555 is a GRPC5D CAR-NK cell derived from iPSCs expressing hnCD16 (> 90%) and IL15RF (> 90%) with CD38 knocked out. Combination of FT555 and anti-CD38 mAb (daratumumab) showed a prolonged persistence compared with CAR-NK cells alone in a multiple myeloma (MM) mouse model (Abstract 1992) [4]. Another study also exhibited the improved anti-tumor efficacy of BCMA/GRPC5D dual CAR-NK cells in MM model (Abstract 3283) [5]. Downregulating immune checkpoint receptor natural killer group 2A (NKG2A) with CRISPR/Cas9 to disrupt the immunosuppression mediated by the tumor microenvironment was shown to significantly improve the antileukemia efficacy of CD33 CAR NK cells in killing assays and AML mouse model (Abstract 1991) [6]. The combination of a Trispecific killer engager (TriKE) capable of binding to the CD16 Fc receptor with IL-15 stimulation and CD33-binding domains and α3 MICA/B CAR-NK cells-controlled leukemia, while progression was observed in α3 MICA/B CAR-NK cells alone under stress (effector: target ratio of 0.25:1) in vitro (Abstract 4623) [7].

In a preclinical study, CD123 CAR-NK cells (5-day OS: 100%) also showed less acute toxicity than CD123 CAR-T cells (5-day OS: 0%) in a mouse model engrafted with human hematopoietic cells, while the antileukemia efficacy was comparable in acute myeloid leukemia (AML) mouse models (Abstract 3279) [8]. Another CAR NK cell therapy for AML showed that CD33/FLT3 CAR-NK cells have exhibited promising antileukemia efficacy (> 90% killing of leukemia cells) in animal models, and the addition of endomucin-inhibiting CARs protected ~ 42% of primary healthy human HSCs and HPCs from cytotoxicity in in vitro assays (Abstract 1978) [9].

Hematopoietic stem cell-derived lymphoid progenitors with Bcl11b inhibition directly differentiate into NK cells rather than T cells, which contributes to the production of stable CAR-NK cells (Abstract 1220) [10] (Table 1).

**Table 1:** Preclinical studies of CAR-NK cells presented at ASH 2022

NK cell source	Diseases	CAR binding target	Improved methods	Targets or combination	Purpose	Abstract number	Reference
iPSCs	Hematological and solid tumors	CD20, HER-2 et al	Structure modification	SLNK12-CAR structure	Efficacy	1983	[3]
iPSCs	MM	GRPC5D	Multiple targets	Daratumumab	Efficacy	1992	[4]
Healthy adult peripheral blood	MM	Dual GRPC5D/BCMA CAR	Multiple targets	/	Efficacy	3283	[5]
Healthy adult peripheral blood	AML	CD33	Multiple targets	Downregulating NKG2A	Efficacy	1991	[6]
iPSCs	AML	α3 MICA/B	Multiple targets	CD33 TriKE	Efficacy	4623	[7]
Healthy adult peripheral blood	AML	CD123	/	/	Safety	3279	[8]
Healthy adult peripheral blood	AML	FLT3 or CD33	Inhibitory CAR	Endomucin	Safety	1978	[9]
HSC-derived lymphoid progenitors	AML	CD123	Genome editing	Incomplete BCL11B suppression	Quantity	1220	[10]

*iPSCs* induced pluripotent stem cells, *HSC* hematopoietic stem cell, *MM* multiple myeloma, *CAR* chimeric antigen receptor, AML acute myeloid leukemia, TriKE Trispecific killer engager

## Clinical trials

A Chinese team presented the initial results of a phase I clinical trial of human umbilical cord-derived CD33 CAR-NK cells for patients with relapsed or refractory AML (Table 2). In 10 evaluated patients, only one patient developed grade 2 cytokine release syndrome (CRS), and no higher-grade CRS occurred. There were no instances of Immune effector cell-associated neurotoxicity syndrome (ICANS) of any grade. All patients with grade 3–4 bone marrow suppression recovered within one month. Regarding antileukemia efficacy, 60% (6/10) of the patients achieved a complete response (CR) 28 days after CAR-NK cell infusion (Abstract 3317) [11]. Another phase I trial of induced pluripotent stem cell (iPSC)-derived B-cell maturation antigen (BCMA) CAR-NK cells is being conducted for MM (Table 2). Neither CRS nor ICANS was observed in 9 patients who received CAR-NK cell infusion (3 received daratumumab as a combination therapeutic agent). One patient treated with 300 million BCMA CAR-NK cells as a monotherapy achieved a very good partial response (VGPR). Two patients achieved a response after 100 million BCMA CAR-NK cells infusion and daratumumab (Abstract 2004) [12].

**Table 2:** Outcomes of clinical trials of CAR-NK cells presented at ASH 2022

NCT number	Disease	Source	Target	Patient number	Preconditioning regimen	Cell dose (10^8^)	Combination	ORR/CR	GVHD	CRS/ICANS	Abstract Number	Reference
NCT05008575	AML	Umbilical cord-derived	CD33	10	FC	6, 12 and 18*	None	60%/60%	None	Grade 2 CRS: 1	3317	[11]
NCT05182073	MM	iPSCs	BCMA	6	FC	1 or 3	None	16.7%/0		None	2004	[12]
				3	FC	1	Daratumumab	66.7%/0				

^*^Three patients received 3 rounds of CAR NK cells (6 × 10^8^, 1.2 × 10^9^ and 1.8 × 10^9^ cells) with an interval of 7 days. Three patients received one dose of 1.8 × 10^9^ CD33 CAR NK cells. In dose group three, four patients received 3 rounds of 1.8 × 10^9^ CD33 CAR NK cells with an interval of 7 days

*AML* acute myeloid leukemia, *FC* fludarabine and cyclophosphamide, *MM* multiple myeloma, *iPSCs* induced pluripotent stem cells, *CAR* chimeric antigen receptor, *CRS* cytokine release syndrome, *ORR* overall response rete, *CR* complete response, *ICANS* immune effector cell-associated neurotoxicity syndrome

Expansion condition, transduction efficiency, and anti-tumor efficacy are the most significant obstacles for CAR-NK cell therapy. In the future, genetically modified methods, preconditioning regimen, cell dose, and combined immunotherapies or hematopoietic stem cell transplant need to be optimized to improve CAR-NK cell therapy, which requires more study to promote the clinical translation of CAR-NK cells.

## Data Availability

Not applicable.

## Abbreviations

CAR: Chimeric antigen receptor
CRS: Cytokine release syndrome
ICANS: Immune effector cell-associated neurotoxicity syndrome
AML: Acute myeloid leukemia
MM: Multiple myeloma
NKG2A: Natural killer group 2A
mAb: Monoclonal antibody
TriKE: Trispecific killer engager
iPSC: Induced pluripotent stem cell
VGPR: Very good partial response
